# Predictive neural signature of internet gaming disorder severity revealed by cross-network connectivity

**DOI:** 10.1093/psyrad/kkag006

**Published:** 2026-01-31

**Authors:** Yi-Hao Hu, Meiting Wei, Xin Luo, Xuefeng Xu, Shuang Li, Anhang Jiang, Guang-Heng Dong

**Affiliations:** Center for Educational Cognitive Neuroscience, Faculty of Education,Yunnan Normal University, Kunming, Yunnan Province 650500, China; Center for Educational Cognitive Neuroscience, Faculty of Education,Yunnan Normal University, Kunming, Yunnan Province 650500, China; Center for Educational Cognitive Neuroscience, Faculty of Education,Yunnan Normal University, Kunming, Yunnan Province 650500, China; Center for Educational Cognitive Neuroscience, Faculty of Education,Yunnan Normal University, Kunming, Yunnan Province 650500, China; Department of Psychology, Beijing Normal University, Beijing 100088, China; Department of Psychology, Shanghai Jiao Tong University, Shanghai 200240, China; Center for Educational Cognitive Neuroscience, Faculty of Education,Yunnan Normal University, Kunming, Yunnan Province 650500, China

**Keywords:** CPM, addiction severity, fMRI, internet gaming disorder

## Abstract

**Background:**

While internet gaming disorder (IGD) correlates with regional brain responses and functional connectivity, the brain network architecture underlying addiction severity remains poorly characterized.

**Methods:**

Using resting-state functional magentic resonance imaging data and addiction severity metrics from 586 participants (443 IGD, 143 recreational game users), we employed connectome-based predictive modeling (CPM) with leave-one-out cross-validation to identify neural networks predictive of IGD severity. The resulting network was evaluated for replicability in independent datasets, with key predictive networks and nodes further analyzed.

**Results:**

CPM identified a replicable addiction severity network. CPM significantly predicted individual gaming addiction scores (*r* = 0.19, *P* < 0.001), with features selected using a threshold of *P* < 0.01. Predictive power primarily derived from internetwork connectivity linking the subcortical, subvisual, and frontoparietal networks. Validation in independent data showed a directional trend (*r *= 0.17, *P *= 0.011).

**Conclusions:**

Individual variability in subcortical–subvisual–frontoparietal network connectivity predicts IGD addiction severity, highlighting these circuits as potential targets for neuromodulation interventions.

## Introduction

Internet gaming disorder (IGD) is characterized by an uncontrollable and excessive engagement in internet-based gaming activities (Dong and Potenza, 2014, 2016; Antons *et al*., 2020. This disorder has emerged as a significant public health concern, particularly affecting adolescents and young adults. Individuals diagnosed with IGD persist in gaming despite being aware of the associated problems, often resorting to deception and evading issues arising from their gaming behavior (Dong and Potenza, 2014, 2016). Given that these behaviors can result in functional impairment (Kim *et al*., 2022; Park *et al*., 2023), the World Health Organization has officially recognized gaming disorder as a psychiatric disorder in the ICD-11 (icd.who.org).

The mechanisms underlying online game addiction are complex, involving psychological, social, and neurobiological aspects. Neuroimaging studies have shown that individuals with IGD exhibit dysfunctions in executive functions, reward systems, and emotion regulation. Gaming addiction is not a dysfunction localized to a single brain region, but rather involves a disruption of the dynamic balance among multiple large-scale brain networks. Functional connectivity (FC) serves as a direct metric for characterizing these abnormal interactions between networks (Seok and Sohn, 2018; Antons *et al*., 2020).

Gaming addiction often manifests as hyperactivity in reward circuits involving the ventral striatum and prefrontal cortex, coupled with reduced functionality in cognitive control networks associated with the dorsal anterior cingulate cortex and lateral prefrontal cortex. FC can quantify the weakening or dysregulation of the connections between these two key systems (Kim and Kang, 2018; Seok and Sohn, 2018). This provides greater explanatory power than measuring the activity of either system in isolation (Dong *et al*., 2011, 2021; Dong and Potenza, 2014, 2016). In terms of functional connectivity, studies have identified abnormal brain functional connectivity in IGD patients compared to typical gamers. Resting-state studies have revealed abnormal functional connectivity in the default, parietal–frontal, and salience networks in individuals with IGD (Yan *et al*., 2021; Gao *et al*., 2025). For example, enhanced amygdala activity is observed in individuals with IGD when exposed to gaming-related cues, with this enhancement correlating with the severity of addiction (Dong *et al*., 2021; Zhou *et al*., 2022). Additionally, weakened frontal–striatal functional connectivity in individuals with IGD has been associated with the degree of addiction (Dong *et al*., 2021; Zhou *et al*., 2022). These findings indicate that IGD is characterized by abnormal functional connectivity in brain regions involved in emotion regulation, executive control, and reward circuits. However, there are certain limitations. First, these studies have not identified comprehensive brain networks associated with the severity of online game addiction, often being limited by the regions of interest (ROIs) they select, which may result in fewer neural indicators of gaming addiction (Garrison *et al*., 2023). Second, the process of ROI extraction is frequently influenced by the researcher’s expertise and experience, introducing a subjective element. Third, the limited neural indicators of gaming addiction hinder a comprehensive understanding of the underlying neural mechanisms of online gaming addiction severity.

Connectome-based predictive modeling (CPM) constitutes a relatively novel methodology within the field of machine learning, with successful applications in the investigation of psychiatric disorders (Song *et al*., 2021; Garrison *et al*., 2023; Zajac *et al*., 2024). CPM incorporates built-in cross-validation techniques to address overfitting by evaluating replicates on new samples, thereby enhancing both rigor and reproducibility. This approach has been employed to elucidate the neural mechanisms underlying gaming addiction and to predict an individual’s level of gaming craving (Feng *et al*., 2024). CPM has proven effective in identifying specific brain networks associated with IGD (Ma *et al*., 2019). While previous CPM studies on IGD have demonstrated feasibility, they often relied on relatively modest sample sizes or specific sub-populations (Seok and Sohn, 2018). This study employs a larger and more diverse sample to enhance the robustness, generalizability, and reproducibility of the identified predictive networks. Furthermore, our goal extends beyond merely identifying correlations to constructing a predictive model of addiction severity based on connectivity patterns, which holds greater translational potential for biomarker development (Seok and Sohn, 2018).

In the present study, we employed CPM to identify networks capable of predicting addiction severity. As studies showed that the severity of IGD is linked to reduced frontal striatal functional connectivity (Dong *et al*., 2021), and with abnormalities in prefrontal cortex (PFC) functional connectivity (Jin *et al*., 2016). The PFC is implicated in cognitive control functions, and abnormal functional connectivity in PFC brain regions may result in impaired cognitive control functions. We hypothesized that we could successfully derive an online gaming addiction severity network and that this network could be generalized across diverse groups of subjects.

## Methods

### Ethics statements

The protocol for this study was approved by the Ethics Committee of Yunnan Normal University, in accordance with the *Declaration of Helsinki*. Written informed consent was obtained from all participants.

### Participants

**Figure 1:**
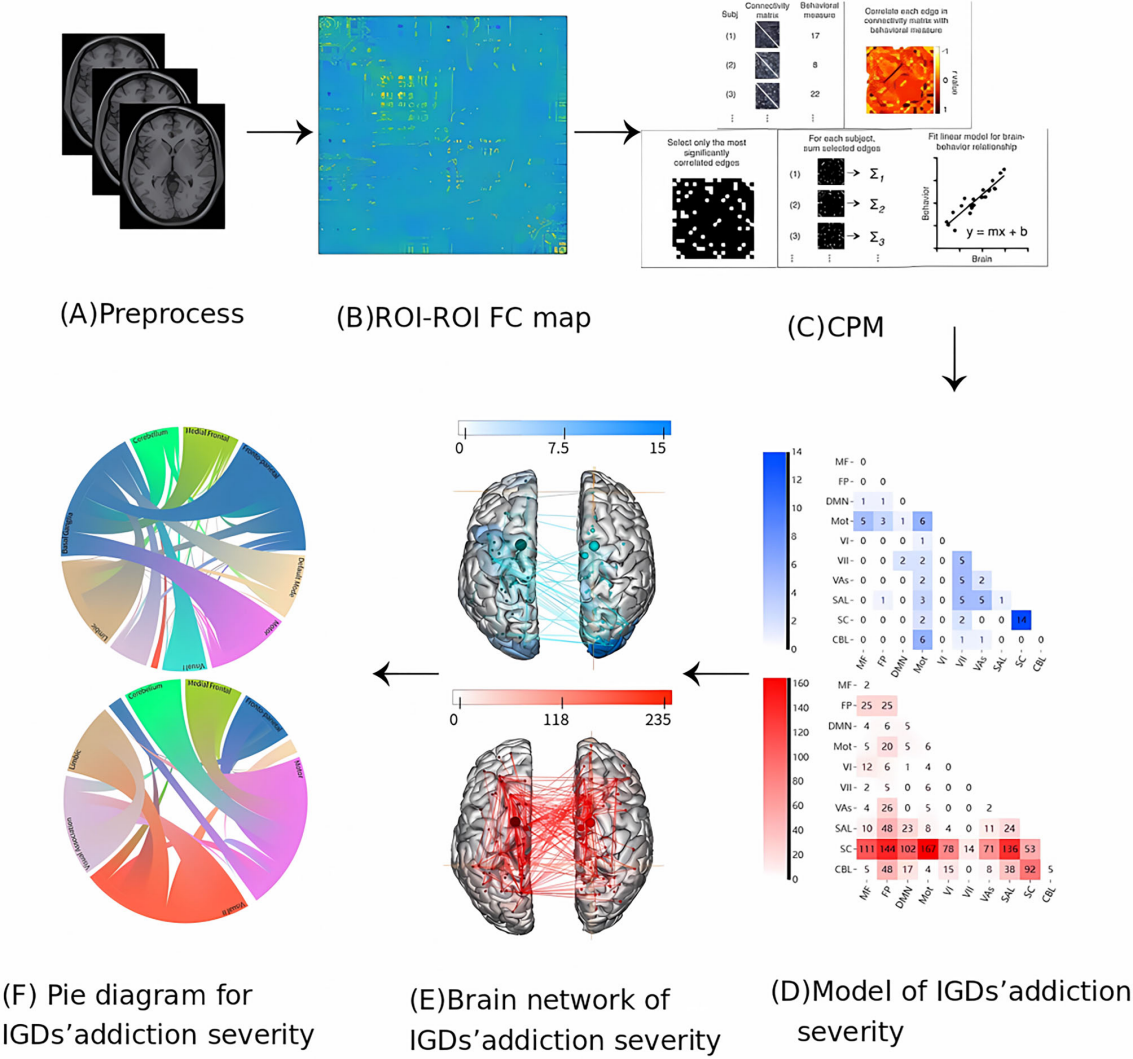
The pipeline of the methodology used in this study. (**A**) Acquisition of resting-state fMRI images. Neuroimaging data analysis was conducted utilizing the DPABI software package. (**B**) Application of the Shen 268 Region of Interest functional template and calculation of ROI–ROI functional connectivity. (**C**) The connectome-based predictive modeling analysis approach (Finn *et al*., 2015). (**D**) Positive and negative networks of addiction severity in IGD. (**E**) Brain networks of severity in IGD. (**F**) Pie diagram for addition severity in IGD.

The whole stuty procedure was shown in Fig. 1. In present study, 443 IGD and 143 recreational game user (RGU) subjects were recruited through advertisement. IGD severity was based on the diagnostic criteria for IGD on Young’s Internet Addiction Test (IAT). After performing a statistical analysis of 586 subjects, the following IAT results were obtained—mean: approximately 60.34; variance [population variance]: approximately 266.19; SD: approximately 16.31. Detailed information is presented in Table 1.

**Table 1:** Demographic information and between-group differences

	IGD	RGU	*t*/χ^2^	*p*
Gender	271/152	139/24		
Age, years	22.3 ± 2.23	22.24 ± 1.39	1.76	<0.05
DSM-5 score	5.815 ± 2.55	3.02 ± 1.64	36.25	<0.001
Craving	54.08 ± 27.60	34.78 ± 18.95	−16.28	<0.001
IAT score	67.515 ± 11.71	36.7 ± 8.64	−32.29	<0.001
Education, years	14.15	14.57	1.42	<0.05

Abbreviations: IGD, internet gaming disorder; RGU, recreational game use; IAT, internet addiction test; DSM-5, DSM-5

proposed criteria.

**Observations on data distribution**

The average range of this dataset is around 60, with SD of about 16. This indicates that the majority of the data points fall within the range of approximately 44–76. A severity threshold of scoring >50 on Young’s IAT was also employed as we have done previously to ensure that individuals with more severe IGD were being studied. Detailed information is presented in Table 2.

**Table 2:** Demographic information and between-group differences in the dataset of external validation

	IGD	RGU	*t*/χ^2^	*p*
Gender	34/23	9/4		
Age, years	20.18 ± 1.635	19.846 ± 0.855	4.38	<0.05
IAT score	71.27 ± 11.90	41.462 ± 7.100	11.69	<0.001
Education, years	13.22	13.65	1.21	<0.05

Abbreviations: IGD, internet gaming disorder; RGU, recreational game use; IAT, internet addiction test.

RGU status was defined as follows: scoring <0 on Young’s IAT and playing online games more than 14 h per week, for a minimum of 2 years, including League of Legends. Participants also underwent assessment using structured psychiatric interviews (MINI) and exclusion criteria for all subjects included: (i) former or current mental/neurological disorders (e.g. depression, anxiety, schizophrenia, and substance dependence) as assessed by a structured psychiatric interview; and (ii) former or current gambling behavior or use of illicit drugs (e.g. heroin or marijuana) or other type of substance addiction (e.g. alcohol).

For external validation, we established a cohort of 70 IGD participants, comprising 27 males and 43 females. Detailed information is presented in Supplementary table 1. Covariates such as gender, age, and head movement were utilized as control variables to mitigate error.

### Imaging

Functional magentic resonance imaging (fMRI) was performed using a 3T Siemens Trio scanner, employing a gradient-echo echo-planar imaging (EPI) T2*-weighted sensitivity-weighted pulse sequence across 33 slices [interleaved sequence, 3 mm thickness, repetition time (TR) = 2000 ms, echo time (TE) = 30 ms, flip angle (α) = 90°, field of view (FOV) = 220 mm^2^, matrix = 64 × 64). Structural images were acquired using a T1-weighted three-dimensional spoiled gradient-recalled sequence encompassing the entire brain (176 slices, TR = 1700 ms, TE = 3.93 ms, slice thickness = 1.0 mm, skip = 0 mm, flip angle = 15, inversion time = 1100 ms, FOV = 240 × 240 mm, in-plane resolution = 256 × 256).

### Preprocessing

Neuroimaging data analysis was conducted utilizing the DPABI software package, a user-friendly plug-in based on SPM12 (http://www.fil.ion.ucl.ac.uk/spm) (Yan *et al*., 2013, 2016). The initial 10 volumes of functional images were excluded to facilitate signal stabilization and participant acclimatization to scanning noise. Subsequent image realignment was executed to rectify any head movement. In the ensuing analysis, 11 participants (six males, five females) were excluded based on criteria of head movement exceeding 2.5 mm maximum translation, 2.5° rotation, or a mean frame shift surpassing 0.2 mm throughout the scans (Power *et al*., 2012; Yan *et al*., 2013, 2016). For normalization of the functional images, participants' structural brain images were initially co-registered with their mean functional images and subsequently segmented.

### Resting state functional connectivity feature extraction

In the present study, network nodes were delineated using a functional brain atlas derived from a graph theory-based parcellation algorithm that optimizes the similarity of voxel-wise time series within each node (Shen *et al*., 2010, 2013). This atlas comprises 268 nodes encompassing the entire brain, including the cerebellum and brainstem. Subsequently, network edges were defined as the functional connectivity between each pair of nodes, calculated as the correlation (Pearson’s *r*) between the time courses of each pair of nodes. The normality of the correlation coefficients was then improved by applying Fisher’s *r*-to-*z* transformation, resulting in the generation of a 268 × 268 symmetric connectivity matrix. This matrix encapsulates the set of edges or connections present within each participant’s resting-state connectivity profile (Shen *et al*., 2010; Finn *et al*., 2015; Rosenberg *et al*., 2016; Shen *et al*., 2017).

### CPM

Initially, a vector of behavioral scores (IAT scores) was correlated with each edge of each participant’s functional connectivity matrix, defined as the correlation of the mean blood oxygen level-dependent (BOLD) signal between specific pairs of brain regions (Figure 1).

**Feature selection**

First, whole-brain functional connectivity matrices were computed for all participants. Then, within the training set, the Pearson correlation coefficient was calculated between the strength of each functional connection (edge) and the IGD severity score (Yoo *et al*., 2018). The purpose of this step is to screen for informative features from the vast number of connections while excluding noise. A correlation threshold (e.g. *P* < 0.01) was set, and connections showing significant positive or negative correlations were retained separately, thereby preliminarily forming the feature sets for the “positive network” and the “negative network.”

**Model construction**

For each participant in the training set, the sum (or mean) of the strengths of all connections within the “positive network” and the “negative network” was calculated, yielding two network feature values. Subsequently, using these two network feature values as independent variables and the IGD severity score as the dependent variable, a linear regression model was fitted (Caicedo-Acosta *et al*., 2021). This step integrates the selected brain network features into interpretable predictive indicators. The separation of positive and negative network feature values facilitates subsequent mechanistic interpretation of their respective roles in promoting or regulating symptoms. Model fitting was performed within a cross-validation framework.

**Cross-validation and performance evaluation**

A 1000-fold cross-validation approach was employed (Pan *et al*., 2022). In each fold, the feature selection and model construction steps described above were repeated, and the resulting model was applied to the test set of that fold for prediction (Pan *et al*., 2022). Finally, the predicted scores and actual behavioral scores from all test set samples were aggregated. This step is used to evaluate the model’s generalizability and predictive accuracy, thereby preventing overfitting to the training data. The primary performance metrics include the correlation coefficient (*r*) between predicted and actual values and its significance (*P*-value), as well as the root mean square error (RMSE). To assess the generalizability of the model, we employed an independent external validation sample set (Dataset 2, *n* = 70) to test the predictive model constructed from the discovery dataset (Dataset 1, *n* = 586). The specific analytical method was as follows: the significant edge sets identified in the discovery dataset (i.e. the positive, negative, and combined networks) and their corresponding model parameters were fixed and directly applied to the functional connectivity data of the external validation sample (Shen *et al*., 2010). Predicted IAT scores were then calculated, and the association strength between these predicted values and the actually observed IAT scores was examined using Pearson correlation analysis (Kumar *et al*., 2019).

The predicted values from the positive network were significantly correlated with the actual IAT scores (*r* = 0.15, *P* = 0.011). In summary, the external validation results indicate that the predictive model constructed in this study—particularly the combined network integrating both positive and negative information—can still effectively predict IGD severity in independent samples. This supports the model’s reproducibility and its potential for clinical generalization (Shen *et al*., 2010; Finn *et al*., 2015; Hong *et al*., 2015).

## Results

After accounting for head movement, age, and gender as covariates, the current CPM analysis effectively predicted IAT scores, with a combination of positive and negative networks yielding (*r* = 0.22, df = 585, *P* = 0.054) with features selected using a threshold of *P* < 0.01, as determined by a permutation test. Notably, the positive network alone significantly predicted the IAT score (*r* = 0.19, df = 585, *P* < 0.001, permutation test) with features selected using a threshold of *P* < 0.01, and the negative network alone predicted the IAT score (*r* = 0.09, df = 585, *P* < 0.001, permutation test) with features selected using a threshold of *P* < 0.01.

**Figure 2:**
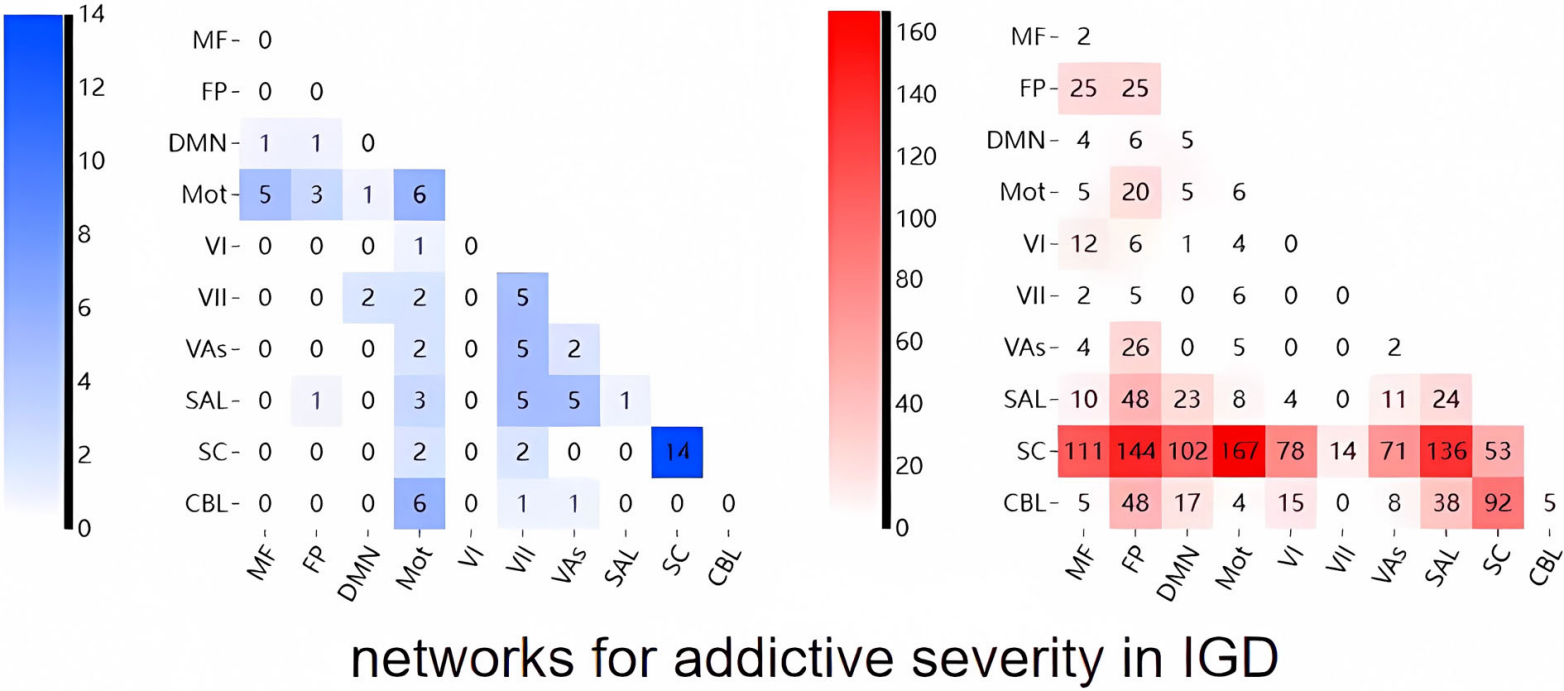
Positive and negative networks for addictive severity in IGD subjects. (**A**) The model selects positive (red lines) and negative (blue lines) networks; a larger sphere indicates more connections for that node. Connections are presented for degree thresholds >30 edges. (**B**) Connections plotted as the number of edges within and between each pair of canonical networks of the positive network. For the matrix on the left, the larger the number of cells, the darker the color, indicating a larger number of edges. Connections are presented graphically on the left. Note that the positive network does not contain the edges of the negative network, and that the connection of a single network and other networks does not contain the edges of the all networks. These five networks are the core of addiction severity in IGD. MF, medial frontal network; FP, frontal parietal; DMN, default mode network; Mot, motor/sensory network; VI, visual a network; VII, visual b network; Vas, visual association network; SAL, salience network; SC, subcortical network; CBL, cerebellum/brainstem network.

Figure 2 presents the positive and negative networks concerning the addiction severity of IGD. In Fig. 2A, in the negative networks, inner connections of the subcortical (SC) network are the core of the severity of online gaming addiction. Inter-connections of the Mot (motor/sensory) network and other networks make up the core of the severity of IGD. Inter-connection of the visual b network and other networks are the core of the severity of online gaming addiction. In Fig. 2B, in the positive networks, inter-connections of the SC network and other networks are the core of the addiction severity of IGD. In Fig. 2B, inter-connections of the Mot network and other networks are the core of the addiction severity of IGD. Inter-connections of SAL and other networks are the core of the addiction severity of IGD. Additionally we sum these networks to more clearly know which networks account for a large proportion. A greater number of connections indicates a higher correlation with the severity of online gaming addiction. Consequently, these networks are ranked to elucidate which exhibit a strong correlation with the severity of online gaming addiction. Among the SC, Mot, FP, DMN, and MF networks, there are significant connections. In Fig. 2B, in the positive network, the SC network ranks first with the highest number of connections (938), followed by the FP network with 353 connections, the SAL network iwith 302 connections, the CBL network iwith 232 connections, and the Mot network with 230 connections. In the negative networks, the Mot network and other networks exhibit the highest number of connections (31), the VII visual network ranks second with 22 connections, and the SC network ranks third with 18 connections. Therefore, the SC, FP, SAL, CBL, and Mot networks are crucial for understanding the addiction severity in IGD. Additionally, the Mot, SC, and VII visual networks are vital for mitigating the addiction severity in IGD.

**Figure 3:**
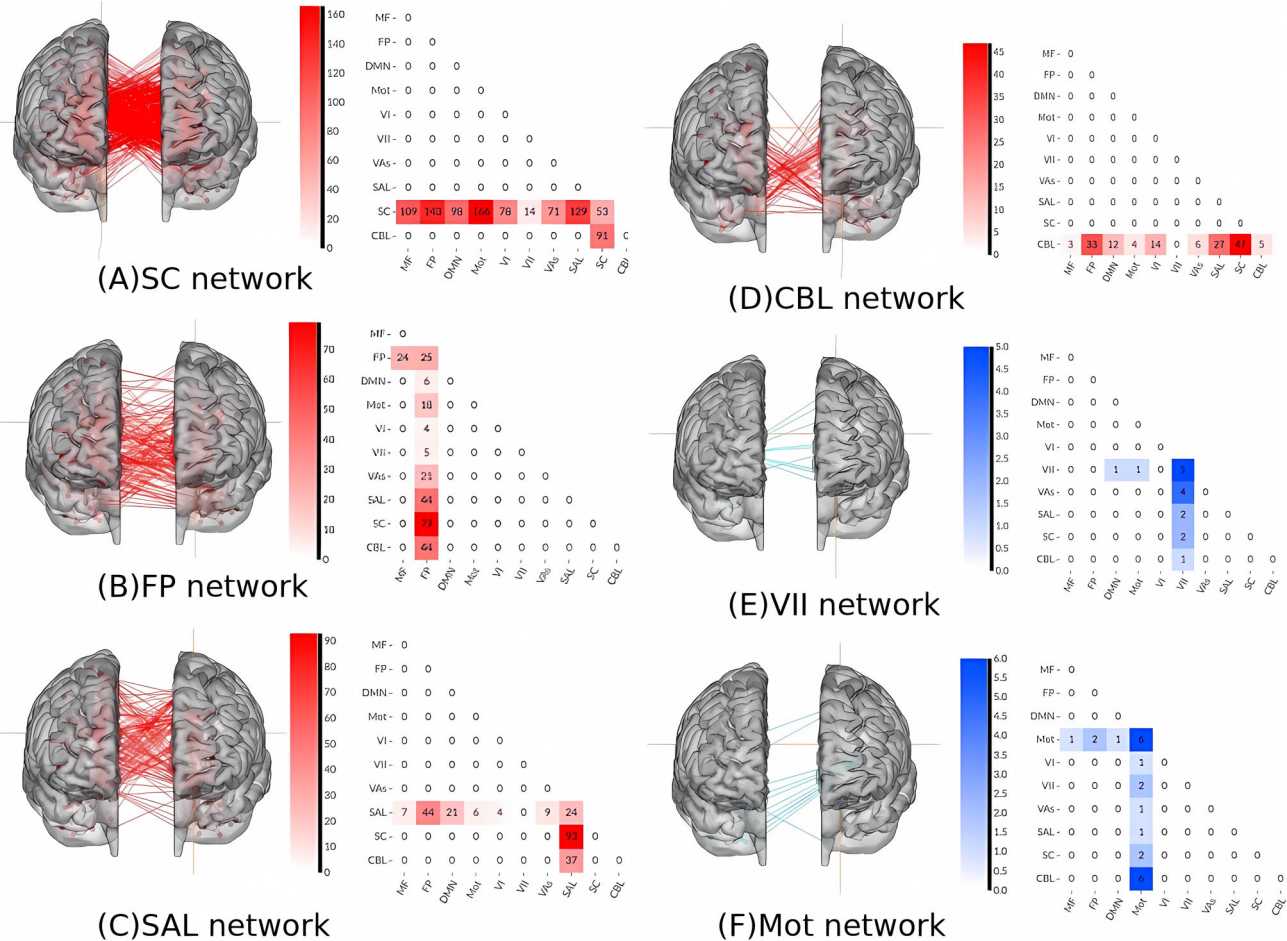
Critical networks for addictive severity in IGD. The six networks for addictive severity in IGD are presented: (**A**) subcortical network, (**B**) frontal parietal network, (**C**) salience network, (**D**) cerebellum/brainstem network, (**E**) visual b network, and (**F**) motor/sensory network. For the matrix on the left, the larger the number of cells, the darker the color, indicating a larger number of edges. Connections are presented graphically on the right. Note that the positive network does not contain the edges of the negative network.

Figure 3 illustrates that an increase in connections correlates with a higher IGD addition severity. To elucidate the significance of each network, the current study reveals that within the positive network, many inter-connection of networks are the core of IGD addiction severity network. Figure 3A indicates that inter-connections of the SC, Mot, FP, and SAL networks are the core of severity in IGD. Figure 3B indicates that inter-connections of the FP, SAL, SC, and CBL network are the core of the IGD addiction severity network. Figure 3C indicates that inter-connectiosn of the SAL, FP, SC, and CBL networks are the core of the IGD addiction severity network. Figure 3D indicates that inter-connections of the CBL, FP, and SC networks are the core of the IGD addiction severity network. In conclusion, inter-connections of the SC, FP, SAL, and CBL networks are the core of positive correlation of severity in IGD.

The current study reveals that within the negative network, many inner-connections of networks are the core of severity in IGD. Figure 3E and 3F indicate that inner-connections of the Mot and VII networks are the core of severity in IGD. In conclusion, inner-connections of the Mot and VII networks are the core of addiction severity in IGD.

The current study reveals that within the positive network in Fig. 3A–D, the SC network exhibits a substantial number of connections with other networks (938). Notably, the SC network maintains strong connections with the Mot, FP, and SAL networks. Similarly, the FP network, within the positive network, demonstrates numerous connections with other networks (353), particularly with the SAL, SC, and CBL networks. The SAL network also shows a significant number of connections with other networks (302), maintaining robust connections with the SC, FP, and CBL networks. Furthermore, the CBL network, within the positive network, has a considerable number of connections with other networks (232), specifically with the SC, FP, and SAL networks. In contrast, within the negative network, the Mot network exhibits a notable number of connections with other networks (31), particularly with the Vas and visual b networks.

### External validation results

**Figure 4:**
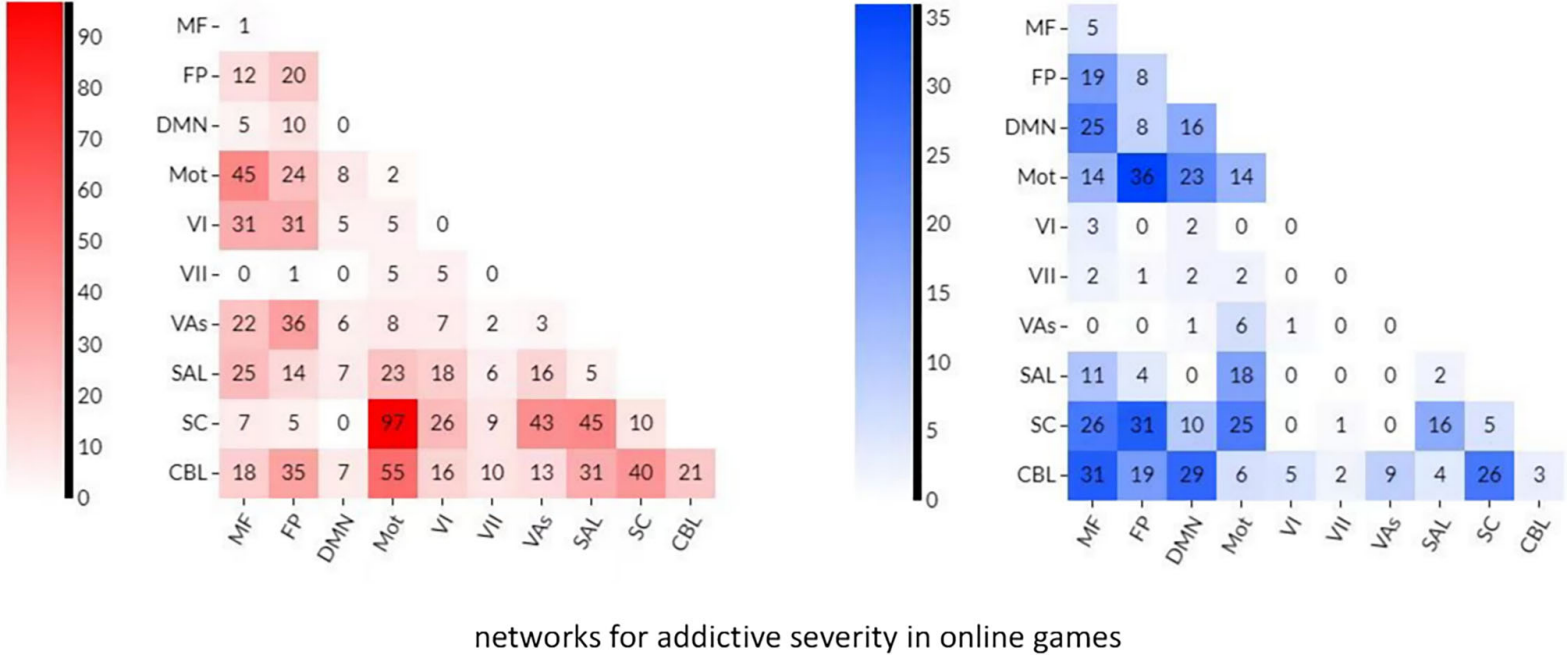
Positive and negative addition severity networks in IGD for the external test. Abbreviations: MF, medial frontal; FP, frontal parietal; DMN, default mode; Mot, motor/sensory; VI, visual a; VII, visual b; Vas, visual assoc; SAL, salience; SC, subcortical; CBL, cerebellum/brainstem. External validation is essential for assessing the replicability of networks related to addictive behaviors in online gaming across various subject groups.

These results suggest that the set of edges and parameters identified in the discovery dataset is especially robust in predicting individual differences in IAT. We built a regression of the model predictions for the independent samples to ensure a relation between two results of the predictive model. We next assessed whether the networks that predicted IAT in the discovery dataset of 586 participants (Dataset 1) generalized to a separate dataset of 70 participants (Dataset 2). Models were run on the external generalizability dataset to assess whether predicted IAT scores generated by models using the set of edges identified in the discovery dataset significantly related to observed IAT scores of those in the generalizability dataset. The results revealed a significant prediction of IAT for the positive network (*r *= 0.15, *P* = 0.011) with features selected using a threshold of *P* < 0.05, and combined network (*r *= 0.17, *P* = 0.01) with features selected using a threshold of *P* < 0.05, but not the negative network (*r* = 0.06, *P* = 0.01) with features selected using a threshold of *P* < 0.05. Note that while *P* < 0.01 for the positive network, the direction of the result suggests that the predicted IAT scores are negatively related to observed IAT scores in the external generalizability dataset, which is not meaningful. These results suggest that the set of edges identified in the positive network are especially robust in predicting individual addiction severity in IGD (Figure 4).

## Discussion

We employ the CPM approach to forecast the addiction severity in IGD. Our study successfully identifies a critical neural network that can predict the addiction severity in IGD. This network serves as a predictive tool for assessing addiction severity in IGD. Collectively, these findings offer evidence that specific neural connections may serve as indicators of addiction severity in IGD. The implications of these results are discussed below.

### Addiction severity in IGD could be predicted by networks

The networks most significantly associated with the severity of addiction in online gaming include the subcortical, subvisual, and frontoparietal networks. The primary nodes indicative of the severity of gaming addiction are the left caudate nucleus head, the right caudate nucleus head, and the right dorsal medial thalamic nucleus. External validation results indicate that the subcortical, cerebellar/brainstem, and motor/sensory networks are the most robust predictors of the severity of gaming addiction. As addiction to online gaming intensifies, the severity network of IGD becomes increasingly pronounced.

### Critical networks for addition severity networks in IGD

Initially, this study identified that the SC network can predict the severity of IGD.

In Fig. 2A, in the positive networks, inter-connections of the SC network are the core of the severity of online gaming addiction. The SC network, serving as the structural hub with the highest number of connections, suggests its potential key role in information integration. Patients with both IGD and tobacco use disorder (TUD) exhibit enhanced connectivity between subcortical and motor networks (Chen *et al*., 2023). This enhanced connectivity may reflect the formation of reward-related motor habits in addictive behaviors. In addition, studies have shown that acupuncture treatment can modulate functional connectivity between subcortical nuclei and frontoparietal networks in addicted individuals, thereby improving symptoms. These regions play a key role in reward, motivation, and executive function. In the negative networks, SC internal networks are the core of the severity of online gaming addiction. It is hypothesized that this connection may be related to cognitive, affective, and reward-related dysfunction in IGD subjects. Thus, the findings suggest that the SC’s internal connections and specific SC-based interactions with other networks may impair cognitive, affective, and attentional processes that are related to the severity of IGD. We hypothesize that this hyper-reactive SC network may override or impair the regulatory capacity of top-down cognitive control networks, such as the frontoparietal network (Zhang *et al*., 2023). The observed predictive importance of SC network-based connections probably reflects this imbalance: salient gaming cues excessively capture neural resources, subsequently disrupting the cognitive, affective, and attentional processes essential for behavioral control (Lee *et al*., 2022). This cycle—driven by enhanced SC network connectivity leading to heightened cue reactivity, followed by compromised cognitive control—may perpetuate compulsive gaming behaviors and directly contribute to the severity of addiction (Lee *et al*., 2022).

In Fig. 2B, in the negative networks, inner-connections of the SC network are the core of the severity of online gaming addiction. The prominence of connections involving the SC network in the IGD severity network is noteworthy. Previous research has demonstrated that recovery from online gaming addiction is associated with functional neural changes within the internal connections of the SC network (Dong *et al*., 2011, 2021; Dong and Potenza, 2014, 2016). This may suggest that the internal connectivity of the SC network is related to addiction severity of IGD.

Second, the study identified that the inter-connection of the FP network and other networks is a predictor of addiction severity of IGD (Dong *et al*., 2011; Dong and Potenza, 2014, 2016). The FP network, serving as the structural hub with the highest number of connections, suggests its potential key role in information integration. The prevalence of connections involving the FP network within the addiction severity of IGD is noteworthy. Furthermore, research has demonstrated abnormal functional connectivity between the FP network and the SAL network in individuals with gaming addiction. For instance, one study reported increased connectivity between the FP network and SAL network in game-addicted individuals compared to healthy controls. This may indicate reduced control of game-related stimuli by the FP network and heightened salience of these stimuli by the SAL network. It has been proposed that the connectivity between the FP network and the motor network (Mot) may be atypical in game-addicted individuals, potentially reflecting the reinforcement of game-related motor behaviors (Chen *et al*., 2024) . It is hypothesized that this connection may be associated with cognitive, affective, and reward-related dysfunctions in individuals with IGD. Thus, we hypothesize that abnormalities in the connectivity between the FP network and other networks may fundamentally stem from a widespread impairment of its cognitive control functions in IGD (Kräplin *et al*., 2021; Kwon *et al*., 2024). This impairment is manifested not only in the deficient regulation of internal reward and affective processes, but also directly in the failure to inhibit responses to specific external cues (gaming-related stimuli) and learned behaviors (gaming operations). This may represent a key neural underpinning for predicting the severity of internet gaming addiction (Kräplin *et al*., 2021; Kwon *et al*., 2024). Additionally, the SC network’s internal connections, as well as SC-based specific interactions with other networks, may impair cognitive, affective, and attentional processes.

Third, in the positive networks, inter-connections of the SAL network and other networks are the core of the severity of online gaming addiction. Within the IGD severity network, there is an unexpectedly high number of connections involving the SAL. Furthermore, research has demonstrated abnormal functional connectivity between the FP network and SAL network in individuals with gaming addiction (Chen *et al.*, 2024) . For instance, one study observed enhanced connectivity between the FP network and SAL network in game addicts compared to healthy controls. This may indicate a reduced control of game-related stimuli by FP and an increased salience of these stimuli by the SAL network. It has been proposed that online gaming addiction is closely linked to altered connectivity in brain networks, particularly between the salience network and other networks such as the executive control network (ECN) and the DMN (Zhang *et al*., 2017). Alterations in these connections may reflect abnormalities in the allocation of attentional resources and cognitive control in addictive behaviors (Zhang *et al*., 2017). Consequently, the findings suggest that SAL-specific interactions with other networks may impair cognitive, affective, and attentional processes associated with IGD severity.

### Critical nodes for addition severity networks in IGD

Highly connected hubs are concentrated in the bilateral caudate nucleus and thalamus. These hubs play key roles in regulating reward processing, motivation, and executive function, and are linked to the severity of IGD (Han *et al*., 2016). The caudate nucleus, part of the basal ganglia, is critical for reward, motivation, and habit formation, processes closely tied to addictive behaviors (Hong *et al*., 2015). As gaming frequency and duration increase, the brain’s reward circuitry (where the caudate nucleus is a core component) remains active for longer periods, potentially strengthening its connections with other brain regions. The thalamus acts as a relay station, receiving sensory and basal ganglia signals and transmitting them to the brain’s cortex. We propose that IGD involves heavy sensory stimulation, making the thalamus a key hub. As addiction worsens, the thalamus may develop stronger connections with other brain nodes.

Replication of the addiction severity network in external data

We find that results of external tests are similar with initial results. Among the positive networks, critical networks of addictive severity in online games are the SC, Mot, CBL, SAL, and FP networks. The critical five networks based on external data are the same as critical networks b 586 subjects. Among the negative networks, critical networks of addictive severity in online games are the Mot networks. Replication of the addiction severity networks are confirmed.

### Limitations

To enhance the reproducibility of the predictive model, as well as for validation and replication purposes, subsequent studies should employ longitudinal validation using resting-state fMRI data and larger sample sizes. This approach will facilitate the elucidation of causality within networks of varying severity. Furthermore, future comprehensive multimodal neuroimaging analyses may significantly deepen our understanding of the neural mechanisms underlying long-term changes in gaming addiction across different severities.

## Conclusions

The current study has successfully identified a critical network for predicting the severity of IGD addiction. This network can be utilized to forecast the severity of IGD. The presence of more connections within these networks correlates with increased severity of online gaming addiction. Furthermore, we employed diverse subjects to test these networks, thereby confirming their scientific validity. The findings further elucidate the neural mechanisms underlying the severity of gaming addiction, identifying brain regions and networks associated with cognitive functions, perception, attention, inhibitory control, and executive function.

## Supplementary Material

kkag006_Supplemental_File
