# Supplementary material for: Predictive neural signature of internet gaming disorder severity revealed by cross-network connectivity
Source: Psychoradiology. 2026 Jan 31;6:kkag006. doi: 10.1093/psyrad/kkag006 (PMC13103295; doi:10.1093/psyrad/kkag006)
Supplement: kkag006_Supplemental_File [file kkag006_supplemental_file.docx]

**Supplementary files for:**

**Predictive neural signature of internet gaming disorder severity revealed by cross network connectivity**

**This file contains:**

**Supplementary Table 1**. **Demographic information and between groups differences**

**Supplementary Table 2. Critical 20 node for addictive severity in IGD (thresh= 0.01)**

**Supplementary Table 3. Ranking networks of addictive severity in IGD**

**Supplementary Figure 1. Positive and negative networks for addictive severity in IGD (thresh=0.01)**

**Supplementary Figure 2. Positive and negative networks for addictive severity in IGD (thresh=0.05)**

**Supplementary Figure 3. The first 20 nodes for addictive severity in IGD**

External validation details

**Supplementary Table 1**. **Demographic information and between groups differences**

|  | Internet games disorder | Recreational game users | *t/χ^2^* | *p* |
| --- | --- | --- | --- | --- |
| Gender | 34/23 | 9/4 |  |  |
| Age(year) | 20.18±1.635 | 19.846±0.855 | 4.383 | *P*<0.05 |
| Internet addition test score | 71.27±11.90 | 41.462±7.100 | 11.69 | *p*<0.001 |
| Education(year) | 13.22 | 13.65 | -1.21 | *P*<0.05 |

**Supplementary Table 2. Critical 20 node for addictive severity in IGD (thresh= 0.01)**

|  | Node | Degree | Lobe | MNI(x,y,z) |
| --- | --- | --- | --- | --- |
| 1 | 260 | 240 | L.Subcortical | 15,3,21, |
| 2 | 122 | 226 | R Subcortical | 15,-3,21, |
| 3 | 128 | 157 | R Subcortical | 6,-9,6, |
| 4 | 263 | 130 | L Subcortical | -6,9,6, |
| 5 | 121 | 92 | R Subcortical | 12,12,12, |
| 6 | 262 | 44 | L Subcortical | 9,24,-3, |
| 7 | 178 | 37 | L Parietal | 9,-66,54, |
| 8 | 135 | 37 | R Prefrontal | -18,18,-21, |
| 9 | 8 | 37 | L Cerebellum | 45,45,-6, |
| 10 | 246 | 32 | L Subcortical | 45,-63,45 |
| 11 | 259 | 31 | L Parietal | -9,9,-9, |
| 12 | 184 | 26 | L Limbic | -54,-45,39 |
| 13 | 220 | 25 | R Prefrontal | 3,6.36, |
| 14 | 19 | 25 | L Temporal | 48,36,15, |
| 15 | 186 | 23 | R Cerebellum | -36,18,-32, |
| 16 | 118 | 20 | R Cerebellum | 36,-45,-54, |
| 17 | 112 | 20 | R MotorStrip | 18,-75,-54, |
| 18 | 30 | 20 | R Prefrontal | 24,12,48, |
| 19 | 14 | 20 | L Prefrontal | 45,12,48, |
| 20 | 152 | 19 | R Parietal | -27,36,-15, |
| 21 | 47 | 19 | L Subcortical | 54,-45,36, |

(L left; R,right)

**Supplementary Table 3.Rank networks of addictive severity in IGD(thresh=0.01)**

| Negative networks(sum) | | |
| --- | --- | --- |
| 1 | Mot | 144 |
| 2 | MF | 136 |
| 3 | CBL | 133 |
| Positive networks(sum) | | |
| 1 | SC | 282 |
| 2 | Mot | 272 |
| 3 | CBL | 246 |
| 4 | SAL | 190 |
| 5 | FP | 188 |

**Abbreviations:** MF, medial frontal network; FP, frontal parietal; DMN, default mode network; Mot, motor/sensory network; SAL, salience network; SC, subcortical network; CBL, cerebellum/brainstem network.

**Supplementary Figure 1. Positive and negative networks for addictive severity in IGD (thresh=0.01)**


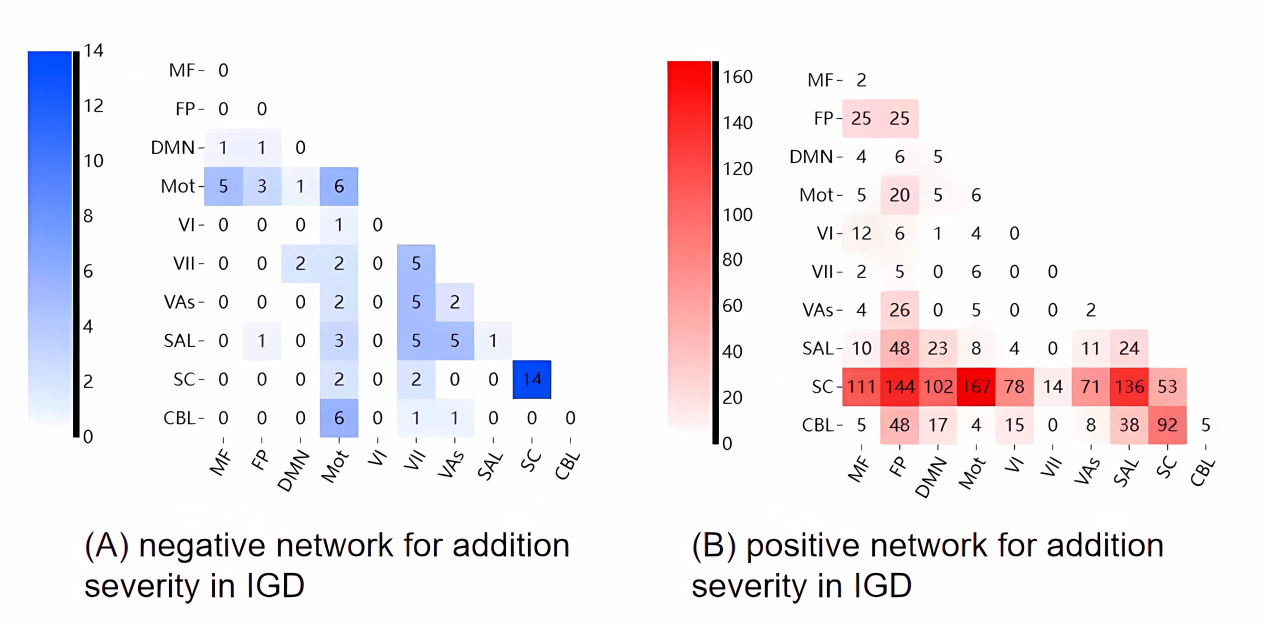


(A)The model selects negative (blue lines) and positive (red lines) networks; a larger sphere indicates more connections for that node. (B) Connections plotted as the number of edges within and between each pair of canonical networks of the positive network. For the matrix on the left, the larger the number of cells, the darker the color indicates a larger number of edges. connections are presented graphically on the right. Note that the positive network does not contain the edges of the negative network.

**Abbreviations:** MF, medial frontal network; FP, frontal parietal; DMN, default mode network; Mot, motor/sensory network; VI,visual a network; VII, visual b network; Vas, visual association network; SAL, salience network; SC, subcortical network; CBL, cerebellum/brainstem network.

**Supplementary Figure 2. Positive and negative networks for addictive severity in IGD (thresh=0.05)
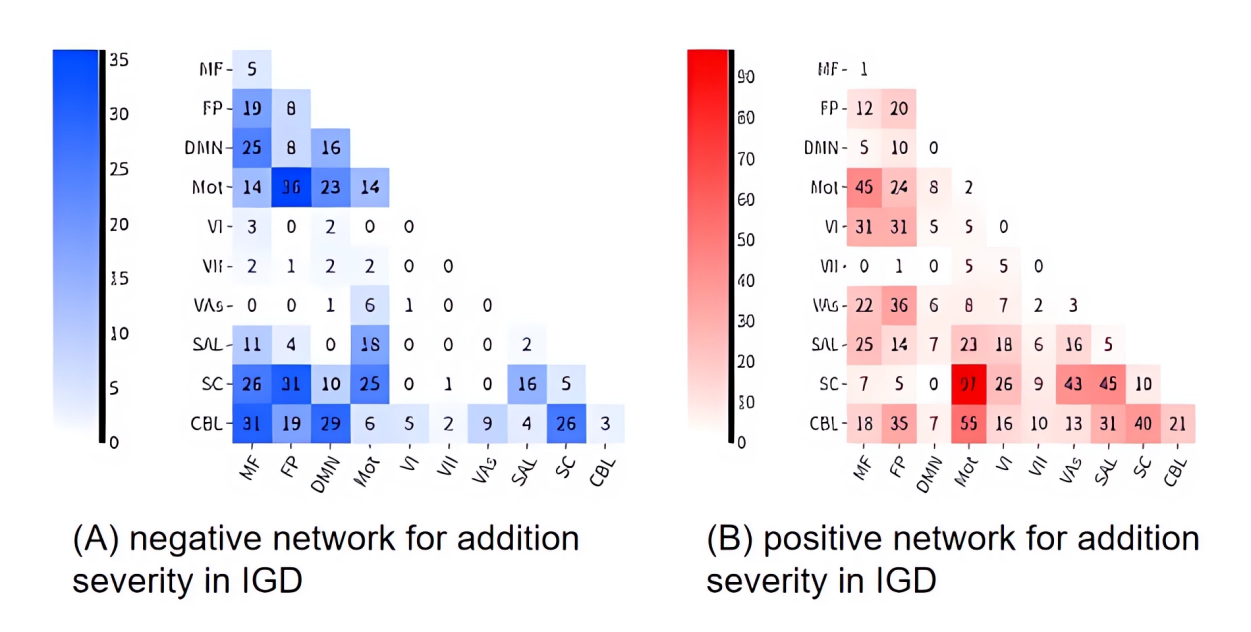
**

(A)The model selects positive (red lines) and negative (blue lines) networks; a larger sphere indicates more connections for that node. (B) Connections plotted as the number of edges within and between each pair of canonical networks of the positive network. For the matrix on the left, the larger the number of cells, the darker the color indicates a larger number of edges. connections are presented graphically on the right. Note that the positive network does not contain the edges of the negative network.

**Abbreviations:** MF, medial frontal network; FP, frontal parietal; DMN, default mode network; Mot, motor/sensory network; VI,visual a network; VII, visual b network; Vas, visual association network; SAL, salience network; SC, subcortical network; CBL, cerebellum/brainstem network.

**Supplementary Figure 3. The first 20 nodes for addictive severity in IGD**


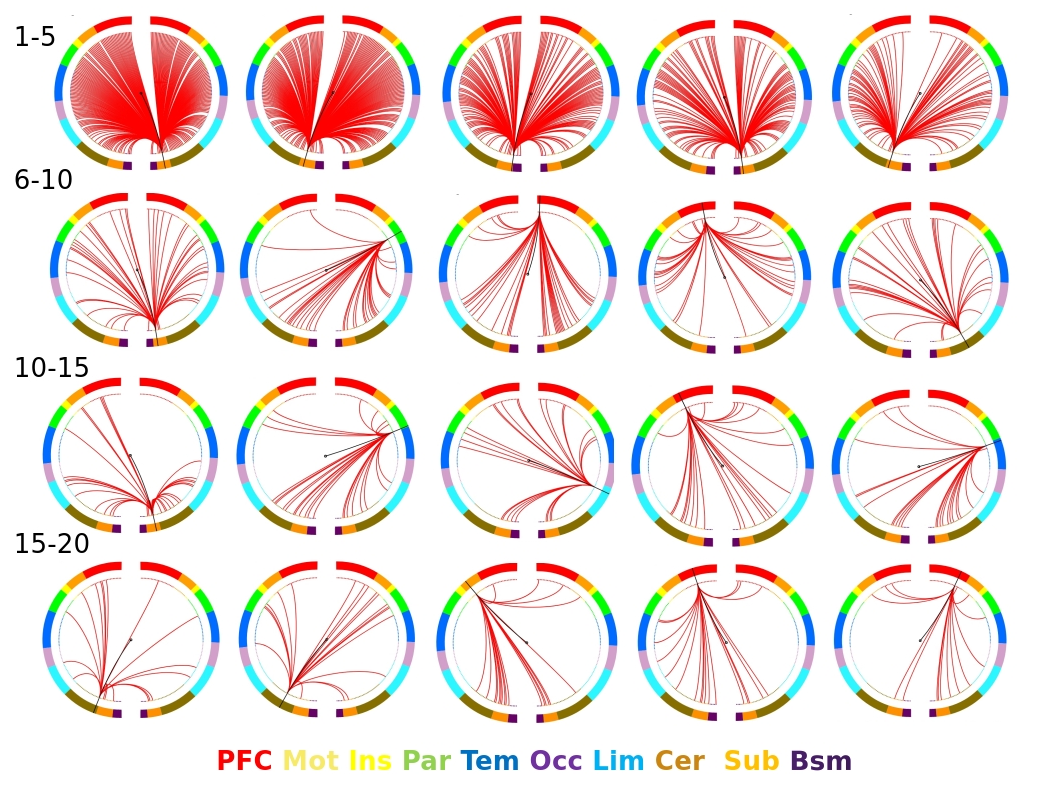


Abbreviations: PFC, prefrontal cortex; Mot, motor;Ins, insula; Par, parietal; Tem, temporal; Occ, occipital; Lim, limbic; Cer, cerebellum; Sub, subcortical; Bsm, brainstem.Different color indicate different brain region. The number of line indicate the number of functional connection.Connectivity patterns of the top 20 nodes with the most connections. Notes: The lobes are prefrontal, motor, insula, parietal, temporal, occipital, limbic, cerebellum, subcortical, brainstem. In Figure 4, the most prominent nodes are associated with subcortical networks and limbic systems. Subsequently, the nodes of significant importance are part of the frontoparietal network, followed by those within the cerebellar/brainstem network. The least significant nodes are associated with the standard DMN network.Detailed information was presented in Supplementary table 2

**External validation details**

In order to verify the reliability of the predictive model based on the main sample, we applied the positive IAT network, negative IAT network, and total IAT network found in Sample 1 as well as the predictive model parameters to Sample 2 to test the generalizability of the predictive model of the brain functional connectome of creative anxiety(Ren et al., 2021). The external generalizability analysis consists of the following three steps: (1) external generalizability feature selection; (2) external generalizability model building; and (3) external generalizability model prediction ability assessment.

Feature selection for external validation: For each sample, the positive IAT network, negative IAT network, and combined IATnetwork found in Sample 1 were used (all three networks were 268×268 matrices, in which the edges contributing to the prediction of IAT were assigned a value of 1, and the edges not contributing to the prediction of IAT were assigned a value of 1). (all three networks are 268×268 matrices, in which edges that contribute to the prediction of IAT are assigned a value of 1 and edges that do not contribute to the prediction of IAT are assigned a value of 0) are multiplied by the 268×268 resting state functional connectivity matrices for each of the samples in Sample 2, thus yielding the positive network, the negative network, and the combined network for Sample 2. In Sample 2, for each sample, the positive network The sum of the functional connectivity strengths of all edges In Sample 2, for each sample, the sum of the functional connectivity strengths of all edges of the positive network is called the positive network strength, the sum of the functional connectivity strengths of all edges of the negative network is called the negative network strength, and the sum of the positive network strength and negative network strength is called the combined network strength. The sum of positive network strength and negative network strength is called combined network strength.

(2) Modeling for external validation: In the externally validated prediction model y=mx + b, positive network strength, negative network strength, and combined network strength are used as the independent variables x, and the behavior "labeling "(IAT score) as dependent variable y, and parameters m and b are from the predictive model of Sample 1.

(3) Assessment of predictive ability of externally validated models: The magnitude and significance of the Pearson correlation coefficient between the true values (IAT scores) and the predicted values (predicted IAT scores) of Sample 2 are used to represent the predictive ability of the externally validated models (the generalization ability of the predictive model of Sample 1). If there is a significant positive correlation between the true values and the predicted values, then the predictive model based on Sample 1 has a good generalization ability (the main results found in Sample 1 can be repeated in Sample 2).
